# Influence of heat treatment on the mechanical properties of CrNi stainless steel orthodontic wires

**DOI:** 10.1590/2177-6709.24.1.068-073.oar

**Published:** 2019

**Authors:** Clariana Hoehne Sepúlveda, Sávio Morato de Lacerda Gontijo, Leandro de Arruda Santos, Alexandre Fortes Drummond, Leonardo Foresti Soares de Menezes, Leniana Santos Neves, Esdras Campos França

**Affiliations:** 1 Restorative Dentistry Department, Division of Orthodontics, Faculty of Dentistry, Federal University of Minas Gerais, Brazil.; 2 Department of Metallurgical and Materials Engineering, School of Engineering, Federal University of Minas Gerais, Brazil.

**Keywords:** Orthodontic, Thermic treatment, Stainless steel

## Abstract

**Introduction:**

The heat treatment of stainless steel wires is a routine clinical procedure adopted by many dentists in order to relieve the stress caused after performing bends in the archwire.

**Objective:**

This study aimed to evaluate the influence of heat treatment of stainless steel archwires with a rectangular section of 0.016 x 0.022’-in.

**Methods:**

For analysis of the dimensional stability, the anterior and posterior dimensions of forty 0.016 x 0.022-in stainless steel orthodontic archwires without heat treatment and 30 days after heat treatment were evaluated. For analysis of the mechanical properties, 12 stainless steel wire segments with the same rectangular section without heat treatment and 30 days after heat treatment were tested through tensile strength and strain tests. To evaluate if there were differences between the anterior and posterior dimensions, the results were analyzed by the Student’s t-test. To compare the tensile strength and strain between the groups, the ANOVA test was used. The level of significance adopted was 95% (*p*< 0.05).

**Results:**

The heat treatment did not stop the expansion of archwires 30 days after their preparation, and there was no statistical difference in the tensile strength and strain tests with and without heat treatment.

**Conclusion:**

From the findings of this study, it can be conclude that the mechanical behavior of heat-treated stainless steel archwires is similar to that of archwires not subjected to heat treatment.

## RESUMO

**Introdução:**

o tratamento térmico de fios de aço inoxidável é um procedimento clínico rotineiro adotado por muitos cirurgiões-dentistas para aliviar o estresse causado após a confecção de dobras no fio. O presente estudo avaliou a influência do tratamento térmico em fios de aço inoxidável com secção retangular de 0,016’’ x 0,022’’.

**Métodos:**

para análise da estabilidade dimensional, foram avaliadas as dimensões anteriores e posteriores de 40 arcos ortodônticos de aço inoxidável de 0,016’’ x 0,022’’ sem tratamento térmico e 30 dias após o tratamento térmico. Para análise das propriedades mecânicas, 12 segmentos de fio de aço inoxidável com a mesma secção retangular sem tratamento térmico e 30 dias após o tratamento térmico foram analisadas por testes de resistência à tração e tensão. Para verificar se houve diferenças entre as dimensões anteriores e posteriores, os resultados foram analisados pelo teste *t* de Student. Para comparar a resistência à tração e tensão entre os grupos, foi utilizado o teste ANOVA. O nível de significância adotado foi 95% (*p*< 0,05).

**Resultados:**

o tratamento térmico não interrompeu a expansão dos fios 30 dias após seu preparo, e não houve diferença estatística nos testes de resistência à tração e tensão com e sem o tratamento térmico.

**Conclusão:**

pelos achados desse estudo, conclui-se que o comportamento mecânico de fios submetidos a tratamento térmico é semelhante ao de fios de aço não submetidos ao tratamento térmico.

## INTRODUCTION

In order to achieve more efficient movement and to prevent damage to the supporting tissue, it is necessary that the orthodontist has knowledge about the structural and mechanical properties of the wires used in clinical practice, since tooth movement is a result of the accumulation of elastic energy and the transformation of this energy into mechanical work. Currently in orthodontics, four basic types of alloys are used: stainless steel; nickel-titanium (NiTi) and its variations (superelastic, thermodynamic and with the addition of copper); beta-titanium alloys, and aesthetic composites.^1^

In 1929, stainless steel wires began to be used in orthodontics and became popular due to its low cost and mechanical properties, such as good formability, biocompatibility, ductility, corrosion resistance, hardness and resilience.^2^

According to the levels of chromium, nickel and carbon, stainless steels can be classified into three types: martensitic, ferritic and austenitic, the first two being basically composed by iron and chromium alloys, and the latter comprising iron-nickel-chromium alloys.^3^ The stainless steel used in orthodontics is the austenitic type.^4^ According to the specification of the American Iron and Steel Institute (AISI), austenitic stainless steels are numbered in the 300 series. Types 302 and 304 are designated as stainless steel (18/8) because the chromium content is 18% and the nickel is 8%. Steels type 302 and 304 have similar compositions, differing only in the carbon content, which is 0.15% in 302 and 0.08% in 304.^5^

Stainless steel wires are used in orthodontic treatment phases that require contour stability of the arches, while keeping the transverse dimensions.^4^

Easy handling due to the good formability or plasticity of stainless steel alloy allows its versatile use in orthodontics, as indicated in different stages of orthodontic treatment.^4^

As with other metals, stainless steel has the atomic diffusion property: at high temperatures, the atomic diffusion rate increases due to the increase in internal energy. Steel wires that are cold worked are not in equilibrium because the atomic migration is negligible.^6^

Cold working can induce significant changes in the microstructure of the wire, which generally increase the strength and hardness of the material, but reduce the ductility and generate residual stresses^7^. To rearrange the atoms of the wire and break the residual stresses, heating is used.^8^

In orthodontics it is generally accepted that the elastic properties of stainless steel (18/8) orthodontic archwires are improved by heat treatment. Thus, heat treatment is performed to relieve stress after performing bends in the archwire, helping to improve its mechanical properties.^9^

However, the heat treatment of stainless steel orthodontic archwires is quite controversial in the literature, due to the different ranges of time and temperature used.^2^ ^,^ ^6^ ^,^ ^7^ ^,^ ^9^ ^-^ ^14^ Supporters of heat treatment claim that the improvement in mechanical properties is due to stress relief, rather than a true transformation or precipitation of carbides.^6^ ^,^ ^11^ Others believe that the improvement obtained is not enough to justify the procedure and that these changes are not clinically noticeable.^9^

The most reliable method to perform heat treatment is using a furnace with inert gas, because the temperature is relatively uniform, preventing oxidation and corrosion due to high temperatures.^15^ Another method of performing this heat treatment is to repeatedly pass the wire through the flame of a lamp until it presents a brownish color throughout its length. It is also common in orthodontic practice to use an electric spot welding machine for heat treatment. However, heat treatment using an electric spot welding machine or lamp does not provide uniform temperature patterns.^16^

Although stainless steel orthodontic archwires have been widely used in orthodontics, the fundamental questions about their mechanical properties after heat treatment still remain conflicting and unclear, which makes this procedure a controversial choice in orthodontics.^9^ Then there has been some discussion on how beneficial the heat treatment is to improve the mechanical properties and the clinical results of stainless steel wire.

As heat treatment is widely used in orthodontic procedures, the aim of this study was to evaluate if its effect on a stainless steel (18/8) 0.016 x 0.022-in rectangular section orthodontic archwires is justified for use in clinical practice. With this objective, the heating was conducted in a furnace, electric spot welding machine or under a lamp; then, tensile strength tests were conducted, and the dimensional stability of archwires with and without heat treatment was evaluated. It was hypothesized that the heat treatment of the steel archwires would not result in improved dimensional stability and mechanical properties.

## MATERIALS AND METHODS

### Materials

The sample used in this study was composed by 40 archwires with 19-mm radius, and 12 segments of 12 cm long of chromium-nickel (CrNi) stainless steel, with dimension of 0.016 x 0.022-in (Morelli^®^, Sorocaba, SP, Brazil).

The heat treatments were carried out using a spot welding machine (Pontomatic Eco, Kernit, Indaiatuba/SP, Brazil), a furnace (Titan, EDG, São Carlos/SP, Brazil) or a lamp. The transverse dimension of the archwires was measured using a microscope (Mitutoyo TM, Aurora, IL, USA), and tensile strength tests were performed in an universal test machine (Instron 5582, Norwood, MA, USA).

### Methodology

To evaluate the effects of heat treatment on the dimensional stability and mechanical properties of the wires, the 40 archwires and the 12 segments of wire were divided into four groups (heat treated in the furnace; heat treated in the welding machine;heat treated under the lamp; and not heat treated).

For the group treated in the furnace, a temperature of 450°C was used for 3 min. The treatment in the spot welding machine used power level 1 for 5 seconds to achieve a brownish color similar to the archwires thermally treated in the furnace at 450°C for 3 min. Heat treatment under the lamp was performed for 27 seconds, to get a brownish color similar to the other heat treated groups.

For the evaluation of dimensional stability, the wires were cut to a length of 18 mm, and from those wire segments 40 archwires were contoured, using as reference the 19-mm radius Interlandi diagram. The heat treatment was performed immediately after making the archwires. The transverse dimension of the archwires was measured under the microscope 24 h after manufacturing the archwires and performing the heat treatment. The measurement points were marked at two distances: the first 6.35 mm from the midline, representing the intercanine width, and the second 19 mm from the midline, representing the intermolar width. These measurements were obtained using a plaster model of the lower arch compatible with an arch radius of 19 mm and with the teeth aligned and leveled. After performing the first measurement, the archwires were again stored for a second measurement, carried out 30 days after the heat treatment.

To evaluate the mechanical properties, the 12 wire segments were subjected to tensile strength testing at a strain rate of 0.0001s^-1^. The wire segments were presented with 12-cm length, and points 3.5 cm from each end were used for mounting on the machine, so the useful segment was 5 cm. The reading of the data was obtained using Blue Hill software.

### Statistical analysis

Data analysis was performed using the Statistical Package for Social Sciences software (SPSS) (IBM Inc., USA) version 22.0. Descriptive statistical analyses were performed to obtain the mean and standard deviation. The normality of the data was verified by the Shapiro-Wilk test. To check whether there was a difference between the intercanine and intermolar dimensions, the results were analyzed by the Student’s *t*-test. For comparison of the tensile strength and strain between the groups, the ANOVA test was used. The significance level of 95% (*p*< 0.05) was adopted.

## RESULTS

**Figure 1:**
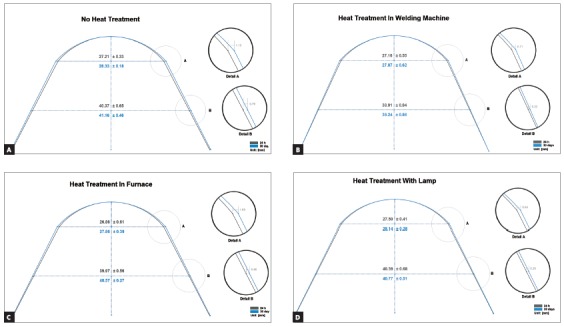
Dimensional stability of 0.016 x 0.022-in rectangular section stainless steel orthodontic archwires, by measuring intercanine and intermolar widths (in mm) 24 h and 30 days after heat treatment, in the following groups: A) no heat treatment; B) heat treatment in welding machine; C) heat treatment in furnace; D) heat treatment with lamp.

Table 1 and Figure 1 shows the results of the dimensional stability of the archwires without heat treatment and, 24 h and 30 days after thermal treatment.

**Table 1:** Dimensional stability of 0.016 x 0.022-in rectangular section stainless steel orthodontic archwires, by measuring intercanine and intermolar widths (in mm) 24 h and 30 days after heat treatment.

Groups	Intercanine width (mm)		p	Intermolar width (mm)		p
	24 h	30 days		24 h	30 days	
No heat treatment	27.21 ± 0.33	28.33 ± 0.18	<0.001	40.37 ± 0.65	41.16 ± 0.46	0.001
Heat treatment in welding machine	27.16 ± 0.55	27.87 ± 0.62	<0.001	38.91 ± 0.84	39.24 ± 0.86	0.007
Heat treatment in furnace	26.86 ± 0.61	27.86 ± 0.39	<0.001	39.97 ± 0.56	40.57 ± 0.27	< 0.001
Heat treatment with lamp	27.50 ± 0.41	28.14 ± 0.28	<0.001	40.39 ± 0.68	40.77 ± 0.51	0.003

Mean ± standard deviation of 10 arches.

The Figure 1 shows that dimensional stability of stainless steel archwires subjected to heat treatment or not subjected to heat treatment suffered expansion in the anterior region (intercanine width) and in the posterior region (intermolar width) in all groups.

Table 1 shows a statistically significant difference between 24 hours and 30 days, with and without heat treatment, for the intercanine and intermolar dimensions; and 30 days after making the archwires, the dimensions were significantly higher, with no stability.

Table 2 shows the results of tensile strength and strain tests of segments of the archwires, with and without heat treatment. Table 2 shows that there was no statistical difference in the tensile strength and strain tests with and without heat treatment. This results indicates that the groups have similar mechanical properties.

**Table 2:** Tensile strength and strain of 0.016 x 0.022-in rectangular section stainless steel orthodontic wire segments, with and without heat treatment.

Groups	Tension (MPa)	p	Deformation (%)	p
No heat treatment	1.836 ± 514	0.514	3.1 ± 1.4	0.488
Heat treatment in welding machine	2.039 ± 118		2.7 ± 0.3	
Heat treatment in furnace	2.162 ± 65		3.1 ± 0.3	
Heat treatment with lamp	1.936 ± 39		2.3 ± 0.1	

Mean ± standard deviation of 3 arch segments.

## DISCUSSION

To perform the heat treatment in the furnace, the temperature used was 450°C for 3 min.^2^ ^,^ ^9^ However, there is no ideal temperature, and the best treatment would be one that confers a brownish color to the archwires.^9^

From the results of this study, a phenomenon known as release of stress was observed, where the dimensional stability of stainless steel archwires subjected to heat treatment (in a furnace, by electric spot welding, or under a lamp) and archwires that were not subjected to heat treatment suffered expansion in the anterior region (intercanine width) and in the posterior region (intermolar width) in all groups 30 days after making the archwires and performing the heat treatment (Table 1). These results show that the tempering techniques used in this study were insufficient to overcome the tension forces of stainless steel contouring. 

With respect to the intercanine width, an expansion of the archwires was observed in all groups, with a greater expansion (1.12 mm) for the control group (no heat treatment) and smaller (0.64 mm) for the lamp group (Table 1). Independent of orthodontic planning, the intercanine distance tends to expand between 1 and 2 mm, and smaller transverse changes in arches are acceptable.^17^ In the present study, the transverse changes did not exceed 2 mm. Although these changes are considered significant in the design of the study, they do not have significant clinical importance. 

With respect to the intermolar width, all the groups analyzed showed expansion of the archwires 30 days after heat treatment, while the control group showed the greater expansion (0.79 mm) and the spot welding machine group the smallest expansion (0.33 mm) (Table 1). As for the intercanine distance, the intermolar expansion was inferior to 2 mm, not presenting clinical importance, despite the statistical difference.

The expansion was a function of time in the anterior and posterior regions of the stainless steel archwires, and this increase in width was more evident in the non-treated archwires, with heat treated archwires being more dimensionally stable.^16^ The intermolar widths, both upper and lower, spontaneously increase from 7 to 18 years of age or until 26 years.^18^ ^,^ ^19^ Other authors advocate a certain freedom in changing the dimensions of the arches, accepting expansions or contractions.^20^

With relation to the tensile strength test performed on the stainless steel wire segments, it was observed that there was no statistically significant difference in the mechanical behavior of all groups (Table 2).

These results suggest that wires which have not undergone cold strain behave similarly whether they were heat treated or not, showing no improvement or even deterioration of their mechanical properties, which corroborates with literature data. In addition, heat treatment is suitable for stainless steel wire and does not change its properties.^9^

The expansion of tensioned U-shaped archwires had a significant increase in tensile strength after heat treatment. This difference compared with the present study may be explained by the fact that orthodontic wires were submitted to cold treatment prior to heat treatment.^11^

The samples used in this tensile strength test did not undergo any type of cold work. 

The majority of austenitic 300 series undergo a martensitic transformation after cold working, and the martensitic phase is responsible for the mechanical resistance.^2^ After annealing, the properties are homogenized and stress generated by the strain is relieved. Thus, the stainless steel wire segments tested in this study experienced no phase change from austenite to martensite, which explains the similar mechanical behavior of the heat treated groups and the control group (not heat treated). 

The maintenance of the mechanical properties of stainless steel wires submitted to heat treatment shows that although it is a practice adopted by many professionals, it is inadvisable, since it has no mechanical benefit, such as the release of stress generated by the conformation of archwires.

## CONCLUSION

This study showed that the heat treatment of stainless steel archwires with 0.016 x 0.022-in rectangular section in a furnace, in an electrical spot welding machine or under a lamp did not prevent the expansion of archwires 30 days after being produced, showing no dimensional stability. Furthermore, the mechanical behavior in tensile tests of stainless steel wire segments that have been subjected to heat treatment was similar to wire segments that were not thermally treated.
